# History of the Royal Medical Society for the Year 1776; with the Memoirs Relative to Physic and Medical Philosophy for the Same Year, Taken from the Registers of the Society

**Published:** 1782

**Authors:** 


IV. *Histoire de la Societé Royale de Medecine. Année* 1776. *Avec les Memoires de Medecine et de Physique Medicale pour la meme Année, tirés des Registres de cette Societé*, i. e. *History of the Royal Medical Society for the year* 1776; *with the memoirs relative to Physic and Medical Philosophy for the same year, taken from the Registers of the Society.* 4to. Paris, 1779. 592 pages, with 16 copper-plates.

THE establishment of a society which is intended to unite physicians of every country in Europe in one general correspondence, will reflect honour on the reign of Louis XVI. This new institution is formed on the most liberal basis, and its views extend to every subject which can in any manner tend to the improvement of medical science.

The

The volume before us, the firſt the Society have publiſhed, is divided into two parts, one of which is allotted to the hiſtory, the other to the memoirs. The former of theſe contains an account of the inſtitution, of the public meetings, prizes propoſed and adjudged, a liſt of the members, eulogies of deceaſed aſſociates, ſome account of the works publiſhed in 1776 by the different members, and ſuch obſervations as are given only in the form of extract. The ſecond part, or memoirs, conſiſts of ſuch eſſays as the Society have thought fit to print at full length.

The preface, which extends through forty pages, contains many judicious remarks concerning the plan of the work, with ſome excellent directions relative to the making of meteorological obſervations for medical purpoſes, the analyſis of mineral waters, and the manner of deſcribing the medical topography of different places, with regard to their climate, ſituation, diſeaſes, &c. Some rules are likewiſe laid down for the deſcription of diſeaſes.

The eulogies of deceaſed members are three in number. The firſt is in honour of JOHN BOUILLET, M. D. ſecretary to the academy at Beziers. He was born at Servian in the neighbourhood of Beziers, March 6, 1690, and died in in his 84th year, Auguft 13th, 1777, leaving two fons and three daughters. The work which gained him the moft reputation was his *Elemens de Medecine pratique*, in two vols. the firft of which was publifhed in 1744. He was likewife the author of feveral articles in the 6th volume of the *Encyclopedie*, and of fome aftronomical obfervations in the *Memoires des Savans etrangers*. It is remarked of him that he had eleven brothers or fifters, of whom nine died after attaining their 80th year, and two furvive him; of the latter, one is 84, and the other 94 years old.

The fecond eulogy is that of JOHN FRANÇOIS LE BEAU, M.D. born at Pont-Beauvoifin in 1721, and educated at Paris and Montpellier, at the latter of which places he graduated in 1747. The year following he was appointed phyfician to the garrifon at Quebec, where he remained till that place was taken by the Englifh. In 1761 he was fent to Louifiana to collect fpecimens of natural hiftory for the king's mufeum; and in this bufinefs acquitted himfelf much to the fatisfaction of M. Bernard de Juffieu. He returned to France in 1774, and the next year was appointed phyfician to the naval hofpital at Breft, and died there of a contagious fever, April 28, 1777.

The

The ſubject of the third eulogy is the celebrated M. De Haller, of whom we have already given ſome anecdotes in our 2d volume.

*Medical Topography.*—This article relates ſolely to Bourdeaux and other places in the province of Guienne.

*Epidemics.*—Under this head we are preſented with, 1. *Some account of epidemic catarrhs*, by Dr. Perkins, phyſician at Boſton, and written at the requeſt of Dr. Franklin. The author firſt deſcribes an epidemic of this ſort which appeared in North America in October 1731, and the following month was obſerved in Ruſſia and Saxony, from whence it ſpread through Holland, Scotland, England, and Ireland. It prevailed at Paris towards the end of January 1732, and in March was rife at Naples. A good account of this diſeaſe is given by Huxham. Dr. Perkins afterwards ſpeaks in a general way of ſimilar epidemics as they occurred in 1745, 1750, 1751, 1753, 1765, 1767, and 1768.—2. *An account of an epidemic which has prevailed for ſeveral years* (1777) *during the autumn, at Villeneuve-les-Avignon.*—The diſeaſe here deſcribed is a remittent fever which owes its ſource to the marſhes in the neighbourhood. It ſeems that the Rhone, on the banks of which Villeneuve is ſituated, has within within these few years shifted its bed, leaving a considerable tract of swampy ground. Previous to this change in the course of the river the town was healthy.—3. *Remarks on a species of epilepsy occasioned by an exanthematic miliary virus.* —These observations are extracted from the correspondence of M. Barailon, physician at Chambon in Bourbonnois, who having observed that Epilepsy is become a frequent disease there since the year 1774, when a miliary fever prevailed in that neighbourhood, considers it as the effect of what he styles a miliary virus. Some cases are related of patients who were cured of their epilepsy by a miliary eruption.

*Diseases of animals.*—This article contains, 1. *An Account* (communicated by the academy at Dijon) *of the disorder which prevailed among the horned cattle at Gessey sur Onche in Burgundy in* 1771 and 1772, and which seemed to be a species of inflammatory fever.—2. *Some remarks*, by M. Adam, physician at Caen, *on a disorder which destroyed the fishes in the river Dives in Normandy.*—3. *Observations on the farcy*, by M. Ialouset, physician at Chatillon sur Soing.

*Practice of physic.*—The first article under this head relates to *schirrus of the Pylorus*, and is written by M. Andry. Several instances are related of this diſeaſe, which we are told is very frequent among the lower ſort of people, who drink a great deal of brandy. In the treatment of this complaint remedies of every kind have hitherto proved ineffectual.—2. *Caſe of an obſtruction of the Pylorus*, by M. Jeanroi. The ſubject of this caſe was a lady, fifty years of age, who died in 1775, after having been for a long time ſubject to inceſſant vomiting, acute pains about the ſtomach, and diarrhœa. On diſſection the pylorus was found ſo contracted as hardly to admit a common probe, and its coats were half an inch in thickneſs. The omentum, meſentery, liver, ſpleen, and pancreas were full of tubercles. The thoracic viſcera were in a ſound ſtate.—3. *Caſe of a contraction of the inteſtines*, by M. de Lalouette.—The patient whoſe caſe is here related was about fifty years old, and had long been addicted to drinking wine and ſpirituous liquors. During the laſt ſix months of his life he complained of dull pains in his bowels, obſtinate coſtiveneſs, and latterly of an inceſſant vomiting of a dark coloured pultaceous matter: his body was opened after death. The ſtomach was found preternaturally dilated at its great curvature, the ſmaller curvature being kept in its ordinary ſtate by a band of

ſchirrous

ſchirrous cellular membrane of the breadth of a finger. The pancreas, which adhered to the ſtomach by means of this band, was likewiſe in a ſchirrous ſtate. The ſpleen was uncommonly ſmall; the liver in its natural ſtate, but the gall bladder was unuſually bulky, being diſtended by a great deal of very viſcid bile. The pylorus, duodenum, and jejunum were in a ſound ſtate; ſome ſlight conſtrictions were obſerved in the ileum; the cœcum was extraordinarily dilated, but the colon abounded with ſtrictures, ſo that in ſome places the canal would hardly admit a ſmall writing pen.—4. *Caſe of apoplexy in a female patient, ſoon after delivery*; by M. Coquerau. —5. *Caſe of a dropſy cured by the uſe of milk*; communicated by Abbé Teſſier.—This account was drawn up by the patient herſelf the Marchioneſs de Grouchy. This lady, at the age of thirty-five years, began to void her urine in ſmall quantity, and to have ſymptoms of anaſarca and aſcites. Diuretics, cream of tartar, Bacher's pills, and other remedies were adminiſtered, but without ſucceſs. At length fourteen quarts of water were drawn off by tapping, and after undergoing this operation ſhe had recourſe to ſome quack medicine, but without finding relief from it. The dropſical ſymptoms continued without any abatement from Auguſt 1776 till January 1777, when to the original complaints were added an inceſſant cough, and bloody ſtools. In this ſtate the patient happening to drink a little milk in order to allay her cough, and finding that it agreed with her, determined to take nothing elſe either as food or medicine. She had not continued long in this reſolution before her urine, which for many months had been loaded, and in ſmall quantity, became clear and copious. Her menſes which had ſtopt for ſix months returned again, and by degrees ſhe recovered her health. It is added that ſhe ſtill finds it neceſſary to adhere to the ſame regimen, ſince whenever ſhe happens to deviate from it her urine becomes leſs abundant.—6. *A caſe of aſcites complicated with anaſarca cured by the uſe of ſorrel boiled in milk*; by M. Favrol, phyſician at Nozeroi in Franche Compté. The ſubject of this caſe was a man 68 years old, whoſe dropſical ſymptoms had reſiſted different remedies. His legs and abdomen were prodigiouſly ſwelled; his urine very high coloured, and in ſmall quantity. Laying aſide the uſe of medicines he had recourſe to milk; boiling a handful of ſorrel leaves in each pint of it. This and ſome bad bread conſtituted the whole of his diet for three months, and at the end of that time he found himſelf perfectly recovered, his thirſt having abated, and his urine begun to flow more copiouſly a few days after he entered on his milk diet.—7. *Obſervations on Madam Nouffer's remedy againſt the Tænia*; by M. Renaud, phyſician to the hoſpital at Barjac. This phyſician has long been in the habit of adminiſtering the fern root in caſes of tænia, but in a manner ſomewhat different from that practiſed by Madam Nouffer, and which has ſucceeded we are told in ſeveral caſes where her's has failed. It conſiſts in the uſe of a clyſter every night, compoſed of two drachms of ſoap diſſolved in a pint of water. The next morning the patient is to ſwallow two drachms of fern root in powder. Theſe remedies are to be repeated every day for five days, and then the patient is to take a purgative bolus compoſed of calomel, jalap, and rhubarb.—8. *Caſe of an abſceſs in the oeſophagus*; by the late M. Macquart. We have here an account of a young lady 19 years old, who after being troubled for about three months with a ſpaſmodic cough began to have a difficulty of ſwallowing, which increaſed ſo faſt that after a very ſhort time ſhe became incapable of taking any nouriſhment by the mouth, ſo that for the ſpace of three months life life was supported solely by clysters. Mercurial and other frictions were employed, but without effect. At length M. Macquart reflecting on the case, and conjecturing that an encysted tumour existed in the oesophagus, and that it might probably be now in a state of suppuration, he resolved to administer some substance which by its weight might occasion a rupture of the sac. For this purpose he prescribed an ounce of crude mercury, mixed with the yolk of eggs, to be swallowed every three hours. This remedy was taken, and the patient soon after she had swallowed the second dose brought up a considerable quantity of pus. From that moment she was able to swallow broth, and by proper care recovered.—9. *An instance of the virtues of the loadstone*; by M. Thouret. The patient whose case is here related had been for eight or nine years subject to a painful affection of one side of his face, which extended to the eye on the same side, and from thence to the temple, and sometimes to the upper part of his head. This pain which returned frequently, and with great violence, having been attributed to carious teeth, he suffered several of his teeth to be drawn, but without the desired effect. Several external applications were employed with the same ill succefs. At length he was advifed to try the ufe of the loadftone. The very firft trial relieved him inftantly, as it were by enchantment, and ever fince that time he has his magnet conftantly in his hand, allaying and difpelling the pain the moment it begins to return, by which means he has rendered his life comfortable. The loadftone he makes ufe of is an artificial one that is capable of fupporting a weight of fix pounds, but he means to procure one that poffeffes twice that power. He obferves that the moment the loadftone is applied to the feat of the pain he experiences a fingular fenfation in the fkin of that part, and a fort of numbnefs is inftantly excited, but that no fuch effects are produced when the loadftone is applied to a part that is free from pain.—10. *Remarks on the virtue of Hoffmann's* liquor anodynus *in the treatment of intermittent fevers*; by M. Defbois, phyfician at Rochefort.—This remedy is recommended in a dofe of fifteen or twenty drops, to be given about an hour before the fit.—11. *An account of a fuppreffion of urine occafioned by a retention of the menfes in the vagina*; by M. Magnan, phyfician at Marfeilles. The girl whofe cafe is here related was 22 years old when M. Magnan firft faw her. She had then never menftruated, but had been long fubject to a pea periodical colic every month; at length her pains became more conſtant, eſpecially about the loins and pubis, and ſhe complained of a ſuppreſſion of urine. Upon examination the vagina was found to be cloſed by a thick membrane which ſeemed to be forced ſtrongly towards the *os externum*. A crucial inciſion being made through this membrane, about two pounds of dark blood were diſcharged, and the patient recovered.—12. M. Poiſſonnier des Perrieres communicates an account he has received from Bourdeaux, relative to the caſe of a M. Herault, an advocate at Blaye, who, five years ago, having had a dangerous fever, loſt the hair from every part of his body, and has lately recovered it again after another fever of the ſame kind.—13. Dr. Strack, of Mayence, in a letter to the Society, adviſes the uſe of a Saturnine Collyrium when the ſmall pox is epidemic. It is to be applied previous to the variolous eruption. He aſſerts that this preventative method has been the means of preſerving the eyes of ſeveral perſons who have had a very confluent ſmall pox. —14. M. Durande, phyſician at Dijon, we are told, has confirmed the efficacy of a mixture of æther and ſpirit of turpentine as a ſolvent of biliary concretions.

[ *To be continued.* ]

